# Vitamin Supplements as a Nutritional Strategy against Chronic Alcohol Consumption? An Updated Review

**DOI:** 10.3390/antiox11030564

**Published:** 2022-03-16

**Authors:** Cristian Sandoval, Jorge Farías, Mauricio Zamorano, Christian Herrera

**Affiliations:** 1 Escuela de Tecnología Médica, Facultad de Salud, Universidad Santo Tomás, Los Carreras 753, Osorno 5310431, Chile; 2 Departamento de Ingeniería Química, Facultad de Ingeniería y Ciencias, Universidad de La Frontera, Temuco 4811230, Chile; jorge.farias@ufrontera.cl (J.F.); mauricio.zamorano@ufrontera.cl (M.Z.); 3 Departamento de Ciencias Preclínicas, Facultad de Medicina, Universidad de La Frontera, Temuco 4811230, Chile; christian.herrera@ufrontera.cl; 4 Núcleo Científico y Tecnológico en Biorecursos (BIOREN), Universidad de La Frontera, Temuco 4811230, Chile

**Keywords:** alcoholic liver disease, vitamin B1, vitamin C, vitamin D, vitamin E

## Abstract

Several studies have shown that blood vitamin levels are low in alcoholic patients. In effect, alcohol use abuse is considered a chronic disease that promotes the pathogenesis of many fatal diseases, such as cancer and liver cirrhosis. The alcohol effects in the liver can be prevented by antioxidant mechanisms, which induces enzymatic as well as other nonenzymatic pathways. The effectiveness of several antioxidants has been evaluated. However, these studies have been accompanied by uncertainty as mixed results were reported. Thus, the aim of the present review article was to examine the current knowledge on vitamin deficiency and its role in chronic liver disease. Our review found that deficiencies in nutritional vitamins could develop rapidly during chronic liver disease due to diminished hepatic storage and that inadequate vitamins intake and alcohol consumption may interact to deplete vitamin levels. Numerous studies have described that vitamin supplementation could reduce hepatotoxicity. However, further studies with reference to the changes in vitamin status and the nutritional management of chronic liver disease are in demand.

## 1. Introduction

In the Diagnostic and Statistical Manual of Mental Disorders (DSM-5) of the American Psychiatric Association, problematic alcohol use is classified as an alcohol use disorder (AUD), which goes from mild to severe depending on the number of diagnostic criteria involved [1]. A range of genetic, behavioral, and environmental variables contribute to alcoholism [2].

Alcoholic drinks are extensively consumed worldwide. Drinking alcohol has negative and positive consequences. The health consequences of alcohol intake vary depending on the amount and pattern of consumption. Although many investigations have provided a correlation between light to moderate alcohol intake and a lower risk of cardiovascular mortality [3,4], some studies found that the link between alcohol consumption and a variety of cardiovascular diseases is ambiguous or negative also at modest intakes [5,6,7].

Ethanol ranks first on the list of abused drugs worldwide. Alcohol use disorder affects about 7.2% of people older than 12 years old, including 6.9% of males and 7.8% of females [8]. It has been described that excessive alcohol drinking promotes the pathogenesis of many diseases, such as cancer, liver cirrhosis, cardiovascular diseases, diabetes, and neuropsychiatric disorders [9,10,11,12].

**Figure 1:**
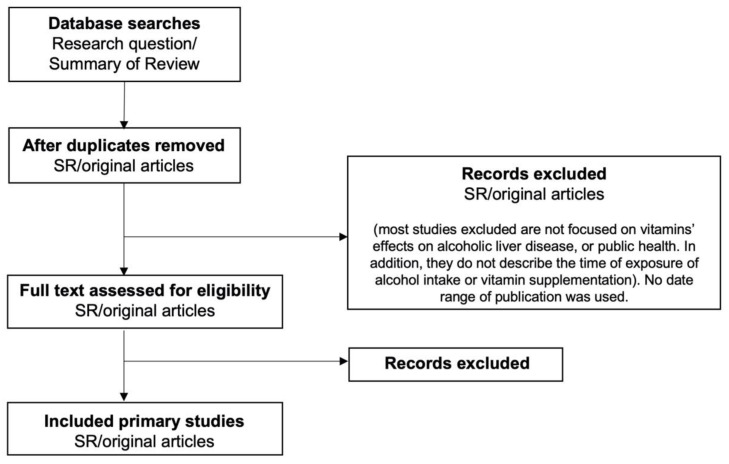
Flow diagram for the review process.

Several associated conditions, such as liver dysfunction, malnutrition, and deficiency in antioxidant vitamins and trace elements, also contribute to the pathogenesis of alcoholic liver disease (ALD). Furthermore, the evidence summarizing the importance of vitamins, their role during ALD, and their possible pathways of action into the disease development is still contradictory. Therefore, the goal of this review is to update the relevance of vitamins and their deficiency during ALD and their potential modes of action during the illness. The flow chart for the study selection process is shown in Figure 1.

## 2. The Pathophysiology of Alcohol Drinking

Ethanol is harmful to the human body and can cause toxicity and death when ingested in excessive amounts. Ethanol metabolism produces an alcoholic fatty liver, alcoholic hepatitis, or cirrhosis [13,14]. The major pathway of ethanol metabolism is the oxidative pathway that involves alcohol dehydrogenase (ADH) present in the cytosol of hepatocytes [15]. This ADH produces acetaldehyde, which is toxic due to its high reactivity and may form DNA or protein adducts [16,17]. Some of the alcohol that is ingested orally does not enter the systemic circulation but may be oxidized in the stomach by ADH and their isoforms. Since the Km of most ADH isozymes for ethanol is low (about 1 mM), ADH is saturated at low concentrations of alcohol, and the MEOS system is activated [18].

Another quantity of ethanol is metabolized by the cytochrome P450 2E1 (CYP2E1) in the microsomal ethanol oxidizing system (MEOS) located within the smooth endoplasmic reticulum of hepatocytes, which leads to lipid peroxidation and to the mitochondrial glutathione and S-adenosylmethionine depletion, producing increased oxidative stress and liver injury [19,20,21]. In addition, fatty acid ethyl esters (FAEE) synthase produces FAEEs via nonoxidative metabolism [22].

Through alcohol intoxication, the CYP2E1-dependent system and the microsomal respiratory chain are the principal sources of reactive oxygen species (ROS) within the hepatocytes. Because of its propensity to metabolize and activate a variety of hepatotoxic substrates in the liver, CYP2E1 is of particular interest. Ethanol, carbon tetrachloride, acetaminophen, and N-nitrosodimethylamine, as well as several hazardous compounds, are among these substrates. [23,24]. In this view, the ethanol-induced activation of cytochrome CYP2E1 appears to be one of the main mechanisms by which ethanol causes oxidative stress. Furthermore, when ethanol is oxidized by CYP2E1, it creates acetaldehyde, a highly reactive molecule that may contribute to ethanol’s toxicity [25]. 

Alcohol-induced liver damage, extracellular matrix changes, and inflammation have all been linked to acetaldehyde [26,27]. Its actions are triggered by the formation of ROS and a redox imbalance (NAD/NADH). It also creates protein clumps in hepatocytes, limiting protein secretion and encouraging hepatomegaly; it combines with dopamine to form salsolinol, which can lead to alcoholism, and it binds to DNA to generate carcinogenic products, such as 1,N 2-propano-2′-desoxyguanosine [12,28].

**Figure 2:**
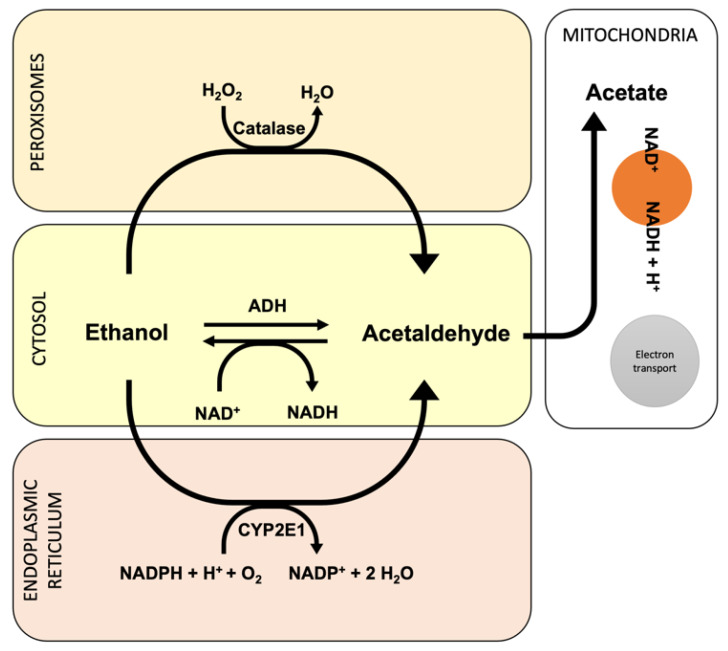
Oxidative pathway involved during ethanol metabolism.

Several research articles have linked alcohol-mediated oxidative stress and ethanol-inducible CYP2E1 with oxidative stress and their toxicity, both in in vitro and in vivo models. In effect, new pathophysiological focuses that could be used against ALD have been described using in vitro studies [29,30]. Nonetheless, hepatocytes’ antioxidant defense can counteract this damage through enzymatic as well as nonenzymatic mechanisms [31,32,33,34,35,36]. Recent clinical trials have examined the efficacy of numerous antioxidants, including S-adenosylmethionine (SAMe) and vitamin E. However, the conclusions drawn by these have been conflicting [31,32,37,38]. As a result, the current study focuses on what we know about antioxidant deficiency and its involvement in AUD and provides suggestions for future trials. Figure 2 shows the oxidative pathway during ethanol metabolism into the hepatocytes.

## 3. Recommended Dietary Allowances

Recommended dietary allowances (RDA) are population statistics, and they represent rough estimates of the average requirement of individuals within a population. However, for most micronutrients, part of the information that is required to accurately calculate the daily intake is either unknown or incomplete. Thus, the recommendations are made based on several assumptions and considerations that could lead to large variations in the eventual RDA [39,40]. In addition, notwithstanding emerging evidence of the remarkable individual differences in the absorption and excretion of vitamins, these values have changed little over the years. It is known that eating requirement values can vary substantially because of several factors, which include genetic polymorphisms, obesity, total energy intake, exercise, and age [41,42,43,44,45,46,47].

The major dietary recommendations for cirrhotic patients are to avoid hepatotoxic substances and to provide enough macronutrient and micronutrient supply in terms of calories, protein, carbs, vitamins supplements, and minerals [48,49].

## 4. Vitamin B 

Previous studies have described that vitamin B (vitamin B1, vitamin B2 and vitamin B6) deficiency in ALD is caused by different factors, such as inadequate dietary intake, increased use of vitamin B, decreased hepatic storage, impairment of intestinal absorption by ethanol, or abnormal metabolism of the vitamins [50,51]. 

Due to decreased hepatic storage, vitamin B9 and vitamin B12 deficiencies can develop quickly in chronic liver illness. However, alcohol consumption affects the metabolism of homocysteine (tHcy) because the enzyme cofactor for the conversion of tHcy to methionine is vitamin B12. Decreased levels of vitamin B12 levels were shown to be adversely connected with tHcy and significantly linked with indicators of alcohol-related liver impairment in recent research [52]. Another research found that individuals with severe chronic liver disease had high vitamin B12 plasma levels but decreased vitamin B9 plasma levels [53]. Conversely, Gibson et al. [54] has shown that two weeks of moderate consumption of alcohol (i.e., red wine, or vodka) increased tHcy and reduced the statuses of both vitamin B9 and B12. In addition, other studies have studied vitamin B status as well [55,56,57]. For example, Van der Gaag et al. [55] showed that type-dependent alcohol had no effect on vitamin B12, but a fall in folate with spirits consumption and an increase in vitamin B6 with all alcohol types were observed. In contrast, Laufer et al. [56] only showed an effect of ethanol on vitamin B12, with no effect on vitamin B9. However, in another study, Beulens et al. [57] showed that beer drinking raised vitamin B6 and appeared to reduce vitamin B12 levels while having no effect on vitamin B9 levels. In this regard, Laufer et al. [56] noted that a lack of vitamins and alcohol use may interact to deplete vitamin B9 and vitamin B12 status and that if nutritional intake matches recommended levels, a decreasing impact of alcohol on vitamin B9 may not be detected. However, further studies are required to clarify the relationship between alcohol consumption and the intake of vitamin B to be able to provide nutritional management strategies for chronic liver disease.

## 5. Vitamin C

One of the many risk factors for vitamin C (including the three forms of vitamin C) and E insufficiency is excessive alcohol intake [58,59]. Vitamin C and E levels are decreased in alcoholics [60]. When compared to those who do not consume alcohol, urine ascorbic acid excretion increased by 47% after acute alcohol consumption of up to 0.58 g ethanol/kg body weight [61]. In effect, pretreatment with vitamin C (doses of 5 g, 1000 mg five times daily for two weeks) significantly improved blood ethanol elimination [62] whereas pretreatment with vitamin C (doses of 2 g, 500 mg four times daily for two weeks) significantly improved alcohol elimination in plasma in the short and long term, implying that vitamin C plays a role in ethanol oxidation [63]. Furthermore, short-term intravenous vitamin C therapy (500 mg/day for five days) significantly improved serum vitamin C levels in chronic alcoholics with hypovitaminosis C [64]. Despite these findings, a previous study indicated that chronic drinkers’ blood levels can take up to three months to restore to normal after taking oral vitamin C supplements [65,66]. 

Hepatocytes metabolize around 90% of ethanol, which is transformed to acetaldehyde by the enzyme ADH. Once the ADH has exhausted its ability to metabolize alcohol, cytochrome P450 isoenzymes take over and convert the molecule to acetaldehyde [67]. This has been found in tissues, including the liver and brain, that have poor ADH activity. By acting as an electron donor and, thereby, unleashing the NAD/NADH pathway, vitamin C is theorized to speed up alcohol metabolism [68]. A positive relationship between ADH activity and leukocyte ascorbic acid concentration has been discovered in people with liver disease [69]. Furthermore, the acetaldehyde produced has been associated with ethanol-induced hepatotoxicity [70,71], and when paired with hepatic CYP2E1 activation, these factors enhance oxidative stress in hepatocytes [12,33,72,73]. On the other hand, vitamin C has been demonstrated to protect against the detrimental effects of acetaldehyde in animal experiments [74]. Given the function of acetaldehyde in the brain’s dopaminergic stimulation of opiate receptors, this could reduce hepatotoxicity and possibly the biochemical basis of addiction [64]. 

## 6. Vitamin D

Calcium homeostasis and bone metabolism require vitamin D to function properly [75]. It is well known for its role in immune response control as well as its anticancer activities [76,77]. Vitamin D deficiency, less than 50 nmol/L of 25-hydroxy vitamin D (25(OH)D) is increasingly being recognized as a global public health issue [78]. According to published studies, the activities and functions of important vitamins and minerals including vitamin B9 and vitamins D, C and E are impaired by chronic ethanol consumption [51,79]. In effect, chronic alcohol consumption has been demonstrated to lower vitamin D levels (inactive vitamin D (25(OH)D3) and active vitamin D (1,25(OH)2D3) as well as cathelicidin/LL-37 expression [80]. 

Immune system deficiency, muscle weakness, osteopenia, osteoporosis, severe upper respiratory tract infections, community-acquired pneumonia, and acute respiratory distress syndrome have all been associated with vitamin D deficiency [81,82,83,84,85]. Furthermore, epidemiologic data linking vitamin D insufficiency to autoimmune disorders, such as multiple sclerosis (MS), rheumatoid arthritis (RA), diabetes mellitus (DM), inflammatory bowel disease, and systemic lupus erythematosus (SLE), have been raised [86]. Vitamin D deficiency, in effect, has been found to hasten the course of existing autoimmune disorders [87]. Reduced immunological function and responsiveness can be caused by lower amounts of inactive vitamin D and active vitamin D. As a result, the frequency of community-acquired and bacterial pneumonia has increased among susceptible populations, such as those with alcoholism [88,89]. Furthermore, in a mouse model of alcoholic myopathy, low vitamin D levels were associated with muscle fiber atrophy [90] where changes in muscular antioxidant enzyme levels may play a key role in the alcoholic etiology.

CYP2E1, an enzyme engaged in ethanol metabolism directly or by creating reactive oxidative metabolites, is implicated in ethanol disruption of enzymes involved in vitamin D metabolism [83,91]. The elevated levels of CYP2E1 seen in broncho-alveolar lavage fluid or liver samples of people with alcohol use disorder are likely due to this [80,92].

## 7. Vitamin E

Antioxidants are necessary for avoiding free radical-induced cellular damage. Vitamin E is a lipid-soluble vitamin that is carried as a component of lipoprotein, and efficiently reduces peroxidation susceptibility both in in vivo and in vitro assays [93,94].

Vitamin E insufficiency has long been linked to ALD [95]. Vitamin E levels in the liver of alcoholics with cirrhosis are frequently low [96]. Vitamin E deficiency, according to earlier research, makes the liver more sensitive to alcohol [97]. In this sense, vitamin E has been demonstrated to have hepatoprotective characteristics in rat models, including membrane stability, reduced nuclear factor-kappa B activation, decreased TNF-α generation, and suppressed hepatic stellate cell activation [12,33,73,95].

There are three histological stages for ALD, and they could be classified into the following: (1) simple steatosis or fatty liver, (2) alcoholic hepatitis (AH), and (3) chronic hepatitis with hepatic fibrosis or cirrhosis [98]. The first-line treatment for severe AH is the administration of corticosteroids [99]. However, some patients with severe AH are refractory to corticosteroids. Nonetheless, Miyashima et al. [100] have reported that vitamin E, as a supplement to corticosteroids therapy, may be a new therapeutic option for these patients. 

By raising ROS and lowering endogenous antioxidant levels, alcohol promotes oxidative stress [101]. In this sense, Prakash et al. [102] have demonstrated that prognostic factors, including the Child−Pugh score and the Model for End-Stage Liver Disease (MELD) score, increased significantly, demonstrating that vitamin E treatment improves short-term mortality more than long-term mortality. In addition, Kaur et al. [103] studied examined vitamin E supplementation in ethanol-treated mice and found that it restored redox state, decreased apoptosis, and lowered oxidative stress markers. However, as compared to the placebo, 1000 IU of vitamin E per day improved serum hyaluronic acid but had no favorable impact on liver function tests or mortality in individuals with mild to severe alcoholic hepatitis [104].

## 8. Possible Mechanisms of Action for Vitamins

Due to ROS being formed naturally, cells have evolved several enzymatic and nonenzymatic ways to protect them [105,106]. In effect, ethanol or its derivatives impairs several of these defensive mechanisms, which could change the redox status, causing the antioxidant cell defenses to be compromised [107]. Nonenzymatic barriers, such as GSH and vitamins, play a key role in several cellular processes. Reduced glutathione (GSH) and vitamins are probably the most important nonenzymatic antioxidants and participate in a wide range of cellular functions. Furthermore, internal redox buffers, such as Hcy, cysteine (Cys), and cysteinyl glycine (CysGly), play an important role in the extracellular redox system [108,109]. Chronic alcohol intake produces altered Hcy metabolism, which leads to fat storage, inflammation, and hepatocyte damage [110,111]. Hyperhomocysteinemia induced by ethanol and linked to oxidative endoplasmic reticulum stress triggers apoptosis and increases lipid production [112].

Previous research has linked alterations in methionine metabolism to ethanol-induced alcoholic liver damage [113,114,115]. In animals and humans, chronic ethanol abuse reduces plasma levels of vitamins such as vitamin B9 and hepatic levels of SAMe [116] and raises plasma Hcy and hepatic S-adenosylhomocysteine (SAH) concentrations [110,114,115]. 

**Figure 3:**
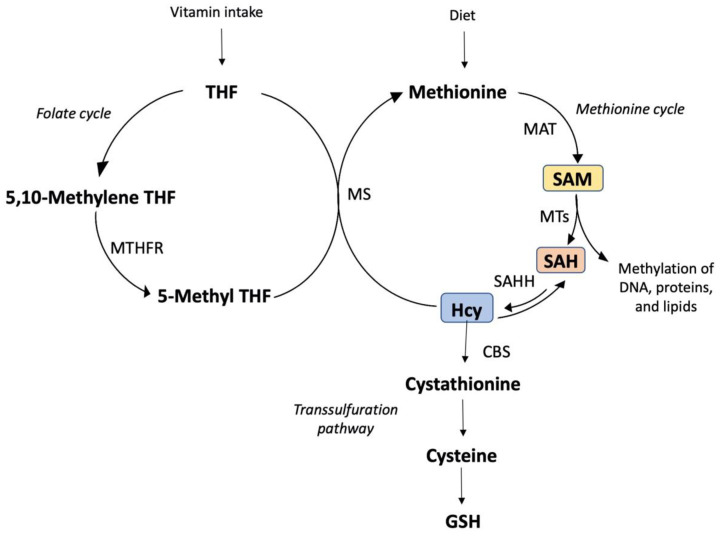
Vitamins and their possible mechanisms of action against the liver injury caused by al-cohol consumption. Tetrahydrofolate (THF), 5,10-methylenetetrahydrofolate (5,10-Methylene THF), methylenetetrahydrofolate reductase (MTHFR), and 5-methyltetrahydrofolate (5-Methyl THF), the initial methyl donor for transmethylation processes, are all involved in vitamin metabolism. 5-Methyl THF and homocysteine (Hcy) are substrates for methionine synthase (MS) in the synthesis of methionine, which is converted to S-adenosylmethionine (SAM) by methionine adenosyltransferase (MAT) in transmethylation processes. S-adenosylhomocysteine (SAH) is a product and inhibitor of methyltransferase reaction (MTs) as well as a substrate for the bidirectional enzyme SAH hydrolase (SAHH), which creates homocysteine or SAH when SAH is in excess. Hcy is metabolized by cystathionine beta-synthase (CBS) and cystathionase to produce cysteine and glutathione (GSH) in transsulfuration processes. It is also important to note that SAM inhibits MTHFR while promoting CBS expression. As a result, a SAM shortage boosts 5-Methyl THF production while lowering 5,10-Methyl THF and GSH production.

There were also associations between both vitamin C and vitamin B9 [117,118] while the molecular basis is unknown. Vitamin B9 is required for the transformation of Hcy into methionine as well as the generation of deoxythymidine monophosphate (dTMP) from deoxyuridine monophosphate (dUMP) [119]. Even though vitamin C consumption has been shown to raise circulation levels of vitamin B9 and lower Hcy levels [118,120,121], the relationship between them remains unknown. Figure 3 summarizes the vitamins and their possible mechanisms of action against the liver injury caused by alcohol consumption.

## 9. Conclusions

The WHO guidelines for withdrawal state the use of multivitamin supplements [122] while another international guideline, published by the World Federation of Societies of Biological Psychiatry, makes mention of the state of hypovitaminosis in the alcoholic but makes no mention of vitamins or their replacement [123].

These antioxidants probably execute their effects through their ability to eliminate reactive oxygen species. However, there is still mixed evidence on the effect of dietary nutrients on the severity of chronic alcohol intake. While the current findings suggest that taking fiber-rich food, consuming water, or eating fat-rich meals could reduce the severity of alcohol hangovers, the last studies have supported the used of vitamins and antioxidants against oxidative alcohol damage. Therefore, new studies are required to elucidate cellular and molecular pathways involved, the mechanisms of action, and the histopathological changes produced after vitamin supplementation.

## 10. Perspectives

Our review updates the existing relationship between vitamins and their mechanism of action during the pathogenesis of ALD. From this viewpoint, we can see that while vitamins perform an important role in the prevention of alcoholic liver disease, other aspects, such as the amount of alcohol consumed, time of exposure (acute or chronic), administration route (oral or intravenous), and administration pre- or post-alcohol drinking, should all be considered when evaluating the effectiveness of vitamin supplementation. Therefore, these variables should be addressed in future studies.
